# What Our Neighbors Say of Us

**Published:** 1884-07

**Authors:** 


### WHAT OUR NEIGHBORS SAY OF US.

THE "ATLANTA MEDICAL AND SURGICAL JOURNAL," (new series, volume 1, No. 1) has made its appearance. It is very handsome, and the interior gives abundant evidence of trained and accomplished editors. The frontispiece is novel and attractive. It is hoped that this Journal will obtain the success which it so richly merits.—*Gaillard's Medical Journal.*

THE ATLANTA MEDICAL AND SURGICAL JOURNAL has changed editors, publishers, paper, type, cover and character of contents. It is much improved in every respect. We wish it a long and successful career henceforth.—*Medical Chronicle.*

NEW DRESS OUT AND OUT.—The ATLANTA MEDICAL AND SURGICAL JOURNAL for March comes to us in a brand new dress. The title-page is very neatly engraved and has a likeness of Crawford W. Long, M. D., the discoverer of anæsthesia. Paper, presswork and contents are all excellent, and the editors and publishers are to be congratulated on the general good looks of this esteemed and long established journal.—*The Southern Clinic.*

The ATLANTA MEDICAL AND SURGICAL JOURNAL has donned a new dress and adorned its cover with a portrait of Dr. Crawford W. Long, the alleged discoverer of anæsthesia. The Journal is not only improved greatly in appearance, but presents an excellent table of contents, and manifests a spirit of enterprise and progress much to be commended.—*Maryland Medical Journal.*

ATLANTA MEDICAL AND SURGICAL JOURNAL.—Monthly; Sixty-four pages. This old and excellent publication starts out with the

March number under a new regimé, and, to judge from the able corps of editors and the ability of those in charge of the mechanical department, the JOURNAL is on the high road to success. The editorials in the first issue relate largely to self, but will repay a perusal. Subscription price $2.50 per year. James P. Harrison & Co., Atlanta, Ga.—*The Drugman.*

